# Boosting of Antibacterial Performance of Cellulose Based Paper Sheet via TiO_2_ Nanoparticles

**DOI:** 10.3390/ijms22031451

**Published:** 2021-02-01

**Authors:** Klaudia Maślana, Anna Żywicka, Karolina Wenelska, Ewa Mijowska

**Affiliations:** 1 Department of Nanomaterials Physicochemistry, Faculty of Chemical Technology and Engineering, West Pomeranian University of Technology, Szczecin, Piastow Ave. 42, 71-065 Szczecin, Poland; Karolina.Wenelska@zut.edu.pl (K.W.); Ewa.Borowiak-Palen@zut.edu.pl (E.M.); 2 Department of Microbiology and Biotechnology, Faculty of Biotechnology and Animal Husbandry, West Pomeranian University of Technology, Szczecin, Piastów 45, 70-311 Szczecin, Poland; Anna.Zywicka@zut.edu.pl

**Keywords:** cellulose, titanium oxide, antibacterial, paper composites

## Abstract

Here, we aimed to boost antibacterial performance of cellulose fibers for paper sheet application. Therefore, TiO_2_ nanoparticles have been used with controlled loading onto the surface of the fibers. A simple and facile composite preparation route based on ultrasound and mechanical assisted stirring has been developed. We tested cellulose paper enriched by TiO_2_ from 1.0 wt% to 8.0 wt%, respectively. Antibacterial performance has been studied against *Staphylococcus aureus* and *Escherichia coli* bacteria. Studies showed that all composites exhibit significant capability to reduce living cells of *S. aureus* and *E. coli* bacteria at least 60%. The simplicity, low cost, and reproducibility of the prepared method indicates the potential to be scaled up for industrial applications.

## 1. Introduction

The development of sustainable or renewable polymer materials is currently a very active research area that receives increasing attention. This is mainly due to the growing demand for materials that are more environmentally friendly than plastics, the production of which is based on petroleum [1]. The main candidate for replacing such materials are natural polymers. The raw materials for them are almost unlimited and do not pose a major threat to the environment due to their simple recycling and biodegradation [2]. The most promising replacement for synthetic polymers is cellulose. This most abundant polymer is widespread, easily degraded, and environmentally friendly, and is therefore a major research target. As the main plant ingredient, cellulose is a sustainable and almost inexhaustible polymer raw material that can meet the growing demand for environmentally friendly products.

Depending on how the cellulose is processed, it can be used in many different fields, such as packaging, clothing, dietary fiber, and many other industrial applications. Unfortunately, unlike other biopolymers (such as, e.g., chitosan [3]), cellulose is not biocidal in nature [4]. Although cellulose itself does not have any antibacterial activity, and even favors the growth of microorganisms on its surface, it is widely studied [5]. Therefore, an important aspect towards the widespread use of this material in various industries is its modification in order to improve its antibacterial properties. It is now very desirable due to the growing public concern about infections, health, and hygiene [6].

Many solutions to this problem have been tried, but still no material has been developed that would provide all the benefits of plastic while at the same time being free from its numerous disadvantages, such as non-biodegradability, creation with the production of toxic gases or harmful pollutions, non-renewable substrate resources, etc. For this purpose, much attention is paid to the modification of cellulose with inorganic compounds, such as zinc oxide, titanium dioxide or copper oxide, and so on [7,8]. The combination of cellulose/metal oxides has been found to form chemically stable composites that exhibit antibacterial properties [5]. Other methods for obtaining antibacterial composites are the addition of nanometric silver, surface modifications, or mixtures with commonly used bactericides [5].

Antimicrobial modification of cellulose fibers can be achieved by three methods: physical mixing, chemical reaction, or final treatment by coating [6]. The ideal agent used to modify cellulose fibers for this purpose should meet a number of requirements. First, the antimicrobial additive should be effective against a broad spectrum of bacteria and fungi. It should also be cheap and low toxicity to users—it should be safe for the skin, non-sensitizing, and non-irritating [6]. Titanium oxide meets all requirements. It is an inorganic compound that is often used in the food industry and has been approved by the US Food and Drug Administration (FDA). It is considered as completely safe, which makes it possible to be used also in food packaging. However, recent research suggests that long-term ingestion may cause serious damage to human health [9,10]. It is a photocatalyst that acts as an oxygen and water absorber and a preservative [11]. Furthermore, the incorporation of this compound in biopolymer packaging may have a number of other potential advantages, such as improved tensile strength, improved heat resistance, reduced permeability, and increased antimicrobial activity [12]. It also has a low cost, biocompatibility, photochemical stability, efficient performance, and environmental friendliness, which further makes it sensible to use this compound to modify cellulose to improve its antimicrobial properties [13]. Although TiO_2_ can inactivate viruses and bacteria and break down particle organics that smell in the air, most of these applications suffer from unsatisfactory quantum yield [14].

The investigation of antibacterial properties of TiO_2_/cellulose have been studied. For example, Li et al. [15] have prepared the cellulose filter paper modified with TiO_2_ by immersing in TiO_2_ sol and further operations. They have tested the antibacterial activity of such materials and found out that TiO_2_/cellulose composite films have little antibacterial effects under either of the dark or UV conditions. The reduction of colony-forming unit (CFU) in dark conditions is 5%, while in UV is 0 against *Escherichia coli.* Another team, Chauhan et al. [16], have prepared cellulose fibers decorated with TiO_2_ nanoparticles using a single-step hydrothermal method. The resulting composites with 3.5% content of TiO_2_ were tested against *E. coli.* After 3 h, the reduction of CFU was 12.5% compared to pristine cellulose. However, after 6 h, the reduction of CFU of modified cellulose had reached 57%. However, there is no report on a simple interaction of cellulose fibers with titania nanoparticles in a wide range of loading in terms of antibacterial or mechanical performance of the prepared paper sheets. The antibacterial activity of TiO_2_ nanoparticles can be explained by several factors. One of the common mechanisms assumes the formation of Reactive Oxygen Species (ROS). Chemical molecules, such as hydrogen peroxide (H_2_O_2_), superoxide anion (O_2_∙-), or hydroxide radical (OH∙) are the most common members of ROS family. These molecules are generated by several stepwise chemical reactions. In this case, the electron-hole pair is generated from the cellulose/TiO_2_ nanocomposite by excitation of electrons from the appropriate band levels through absorption of light and these electron-holes produce ROS. The amount of generated ROS depends on threat of migration, generation rate, and energy level of the excited electron-hole pairs. According to the above, hydrogen peroxide molecules are generated, which can damage DNA, proteins, amino acids, and carbohydrates in bacteria cells [17]. By introducing remarkable photocatalytic properties of titanium dioxide, different mechanisms can be involved. In this case, cellulose/TiO_2_ nanocomposites accept light irradiation as the source of photon energy. If the energy absorbed from photons is higher than the bandgap energy, the electrons (e^−^) from the valence band are stimulated and excited to the conduction band. This step results in generation of a positive hole (h+) in the valence band and produces good electron holes on the catalytic material surface. Different ROS via the following reactions could be produced [18,19]:

(1) cellulose/ TiO_2_ + hυ → e^−^ + h^+^

(2) H_2_O + h+ → OH∙ + H^+^

(3) O_2_ + e^−^ → O_2_

(4) O_2_^−^ + H^+^ → OH_2_

(5) e^−^ + OH_2_∙ + H^+^ → ROS

Another mechanism includes the relationship between the antibacterial properties and the morphology of the active material. In this case, the antibacterial activity highly depends on the size and shape of nanoparticles [20]. The smaller size of nanoparticles favors the ease of passage through the bacteria cell walls and disturbs the metabolic pathway of bacteria by influencing the shape and function of the cell membranes. This can cause the dysfunction of mitochondrial, DNA damage, or overall, the death of bacteria.

The last mechanism is based on the negatively charged cell walls. This fact results in easy attachment of released Ti^4+^ ions from cellulose/TiO_2_ nanocomposites due the interaction with the cell membrane. Metal ions show, then, interaction with the functional groups of nucleic acids and proteins, such as: -NH (amino), -COOH (carboxyl), and -SH (mercapto) groups, affecting their normal physiological processes of bacteria, damaging cell structure, and deactivating the overall enzyme activity [21,22]. In our work, we present a simple and facile route to prepare a cellulose-based composite with TiO_2_ nanoparticles. The composites with nanoparticles loading from 1 to 8 wt% have been prepared and characterized in details by means of various microscopic, spectroscopic, thermal tools. In addition, antibacterial tests have been described. The results shown satisfactory results at the level of at least 60% of reduction of living bacteria. To the best of our knowledge, there is a lack of similar systematic study into the effects of TiO_2_ on the antibacterial properties of paper composites. Here, we describe a simple method of preparation paper sheets on a machine imitating conditions similar to large-scale production. Therefore, the results of this study could be easily scaled-up.

## 2. Results and Discussion

**Figure 1:**
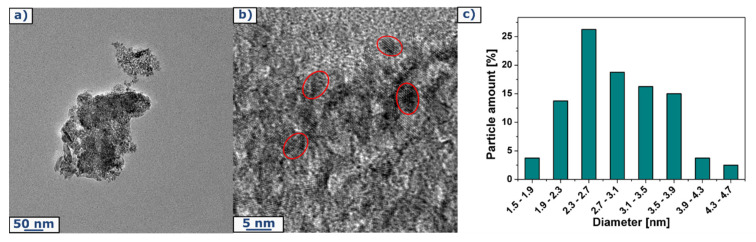
(**a**,**b**) Transmission Electron Microscope image of TiO_2_ and (**c**) corresponding particle size distribution chart.

Morphology of TiO_2_ was first observed by transmission electron microscope (Figure 1a,b). It shows the round-structured nanostructured TiO_2_. Cleary, the nanoparticles have a visible tendency to agglomerate. According to TEM images, particle size distribution has been analyzed. Figure 1c presents averaged nanoparticle size corresponding to 100 measurements of single particles’ diameters. It is clearly shown that TiO_2_ has nanometric structure with most particles with a diameter in the range of 1.9–3.9 nanometers.

**Figure 2:**
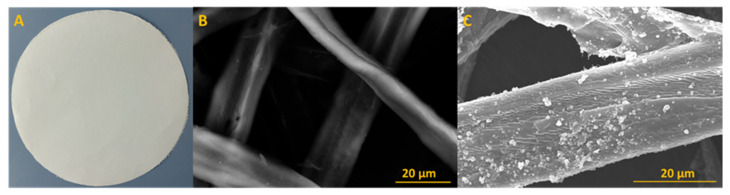
(**a**) Obtained paper disk form Rapid apparatus and (**b**) Scanning Electron Microscope image of reference paper sheet and (**c**) paper sheet with TiO_2_ 8%.

All prepared cellulose paper sheets (showed at Figure 2a) with a diameter of 20 cm possess a paper weight about 80 g/m^2^. SEM images are presented at Figure 2b,c. The image of reference sample (Figure 2b), which is a cellulose sheet without any additives, exhibits pristine cellulose fibers, without any particles on the surface. The paper sheet with 8 wt% of additive is illustrated in Figure 2c. It is clearly seen that small particles are deposited on the surface. This is assigned to deposition of titanium oxide agglomerates on fibers surface.

**Figure 3:**
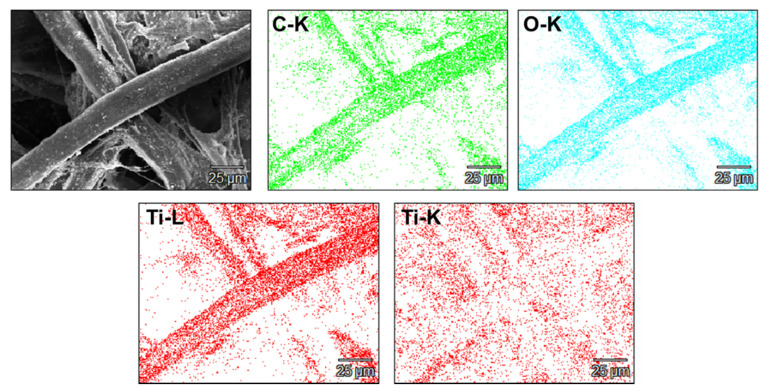
EDS mapping of cellulose fiber modified with TiO_2_ 8%.

Additionally, EDS analysis (as SEM mode) was performed for paper sheet modified with 8 wt% of TiO_2_ (Figure 3). According to the results, it can be concluded that TiO_2_ was uniformly distributed over the cellulose fibers. The composite sample with 8 wt% TiO_2_ is presented as representative here due to the most noticeable changes taking place on the cellulose fiber. A small amount of silicon impurity is present in the sample. Due to the fact that the cellulose used is not chemically pure and may have contaminations resulting from the sample production line, this element is omitted in further work.

**Figure 4:**
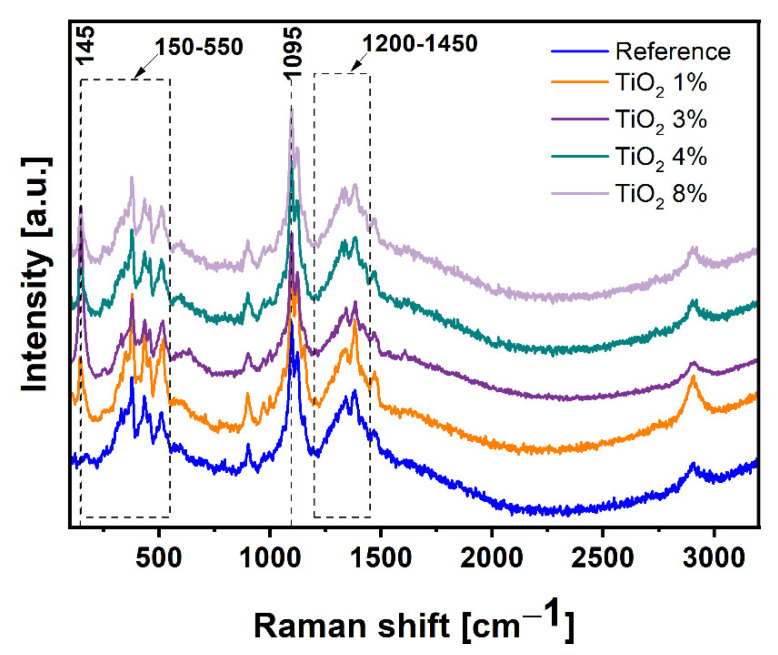
Raman spectra of all samples.

Raman spectra of all samples, which are referring to the cellulose sheet and cellulose sheet decorated with 1 wt%, 3 wt%, 4 wt%, and 8 wt% TiO_2_, are presented in Figure 4. The peaks obtained for all samples are typical for cellulose-I structure [23]. All plots exhibit peaks located near 1095 cm^−1^, which is characteristic for cellulose materials and corresponds to C-O ring stretch modes within cellulose backbone [24,25]. A number of peaks located in the region ranging from 1200 to 1450 cm^−1^ are attributed to modes involving considerable coupling of methine bending, methylene rocking and wagging, and C-O-H in-plane bending vibrations [23]. The main difference between all four patterns is the appearance of a peak located at 145 cm^−1^, which can be assigned to incorporation of titanium dioxide to structure and is in accordance to literature [26,27]. Lack of other peaks typical for TiO_2_ in the region near 390–650 cm^−1^ can be explained by overlapping of cellulose peaks for which skeletal bending modes of C-C-C, C-O-C, O-C-C, O-C-O dominate in the region of 150–550 cm^−1^ [28].

**Figure 5:**
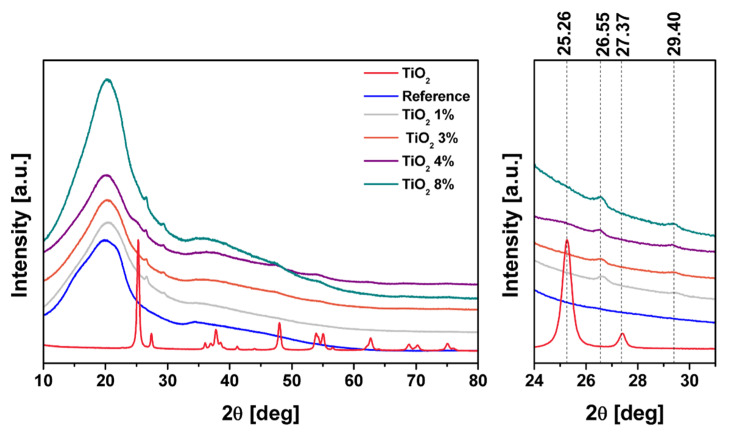
X-ray diffraction spectra of all samples.

Figure 5 reveals XRD spectra of pure TiO_2_, cellulose paper as a reference, and its composites. All samples were ball-milled before analysis to increase the visibility of some peaks. As it can be easily seen, the difference in the crystallinity of the samples is noticeable due the difference in the sharpness and width of peaks in pristine TiO_2_ compared to other samples, indicating more amorphous structure of cellulose and its composites. Additionally, all four patterns for cellulosic materials are similar. The main difference is the appearance of two small peaks located at 26.55 and 29.40 degrees for composite samples. This can be assigned to the presence of TiO_2_ in the structure. These peaks correspond to two characteristic peaks for anatase and rutile phases of TiO_2_, which occur at 25.26 and 27.37 degrees. The shift of 2θ value towards higher values can be explained by the change in the lattice parameters of TiO_2_, which occurs as a result of composite preparation [29]. According to Braggs’ law, the higher 2θ angles, the lower the d-spacing value. XRD spectra of pure TiO_2_ indicates the presence of two crystal structures of titanium oxide: anatase and rutile. The presence of anatase form is confirmed by the presence of peaks 2θ = 25.26° (101) and 2θ = 48.02° (101). Peaks at 2θ = 27.36° (110) and 2θ = 53.91° (110) indicate the rutile form [30]. The percentage of the phases was calculated in accordance to Spurr and Meyers equations [30,31,32]:

X_A_(%) = 100/(1 + 1.265 I_R_/I_A_) (1)

X_R_(%) = 100/(1 + 0.8 I_A_/I_R_) (2)

where X_A_ and X_R_ are the percentage weight of anatase and rutile, respectively, I_A_ is the intensity of anatase phase at 2θ = 25.26°, and I_R_ is the intensity of rutile phase at 2θ = 27.36°.

According to above Equations (1) and (2), the rutile percentage mass is equal to 76.28% and 23.50%, respectively.

**Figure 6:**
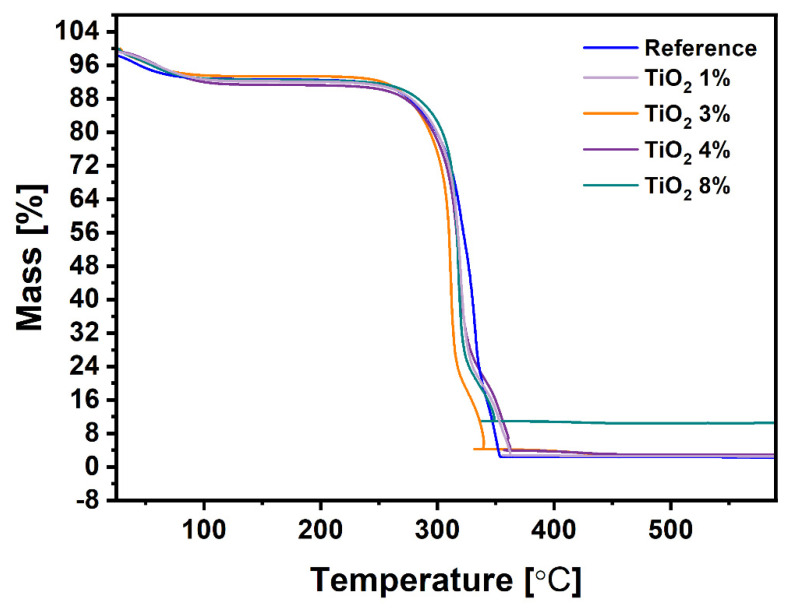
Thermogravimetric analysis pattern for all samples.

Thermogravimetric analysis was involved to determine the thermal stability of all samples. Each sample shows similar thermal decomposition (Figure 6). The weight loss in the temperature range to 100 °C is due the water evaporation. Clear degradation of cellulose materials starts at around 300 °C. The TGA curves indicate that near 98% of pristine cellulose is thermally degraded. For composites containing 1 wt%, 3 wt%, 4 wt%, and 8 wt% of TiO_2_, these values are equal to 97.5%, 97.1%, 97%, and 90%, respectively. The increase of residue after the measurement is in correlation with the mass of TiO_2_ incorporated to the cellulose mass.

Additionally, the actual content of additives has been determined in accordance to the International Organization of Standard ISO 1762:2001 for determination of residue (ash) on ignition at 525 °C. Basically, properly prepared samples were heated with specific heating speed to 525 °C and held at this temperature for 3 h. The mass of obtaining ash was assigned to content of TiO_2_, since all cellulose fibers should have been burned under these conditions. Calculated percentages of TiO_2_ for all samples have been collected in the Table 1. For comparative reasons, the mass values at 525 °C determined by TGA were also presented. There is a noticeable difference of residual value for each sample; however, the trend is maintained. This is due to more precise measurement conditions according to ISO 1762:2001 and the amount of sample required for analysis. A significant change of additives added to the cellulose pulp and ash, which is TiO_2_ remaining in the sample, can be observed. This phenomenon is caused by small particles of TiO_2_ passing through the mesh of the Rapid apparatus sieve, which leads to incomplete retention of TiO_2_ on the cellulose fibers. A small amount of reference ash can be attributed to the contamination from the production line.

**Figure 7:**
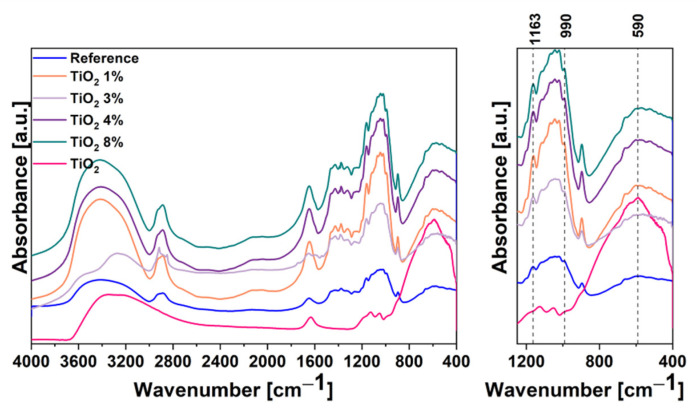
The Fourier transform infrared (FTIR) spectroscopy spectra of the TiO_2_, cellulose, and its composites.

FTIR was involved in order to determine chemical composition of samples (Figure 7). Additionally, FTIR spectrum was obtained for TiO_2_ for comparative purposes. Firstly, a small amount of ball-milled cellulose paper sheets (~2 mg) was precisely mixed with KBr (~200 mg) by grinding. Then, KBr-based 13 mm pellets were formatted by hydraulic press. The background KBr was subtracted from the spectrum of each sample. It can be seen that for each spectrum, a broad peak near 3450 cm^−1^ is observed and can be attributed to O-H stretching [33]. A signal occurred at 2915 cm^−1^ for reference and composite samples were assigned to -C-H stretching [34]. The lack of that peak for pure TiO_2_ sample is reasonable because of the absence of carbon in the structure. The main difference between all patterns is visible at the region of 1300–450 cm^−1^. The wide and very intense peak can be observed for pure TiO_2_ sample in the range of 400–800 cm^−1^ [35]. Additionally, with increasing TiO_2_ content in composite samples, the signal intensity in this region increases, which proves that TiO_2_ content is higher. Around 667 cm^−1^ small signal can be observed for the reference spectrum. This signal can be attributed to the out-of-plane C-OH bending in reference and composite samples [36]. The peak located at 898 cm^−1^ is characteristic for β-glycosidic linkage between the glucose unit and can be observed for each sample, except for TiO_2_ [37]. The peak located at 1376 cm^−1^ for reference and the composite sample can be attributed to the C-H group in the glucose unit and is characteristic for cellulose materials [37]. For each sample, two peaks located at 3367 cm^−1^ and 1634 cm^−1^ are observed and can be attributed to the stretching and bending vibrations of hydroxyl group, respectively. These signals are associated to the physiosorbed water molecules to the surface of samples [38]. There is one broad peak located at 590 cm^−1^ which can be attributed to Ti-O-O vibration modes [39]. This proves that TiO_2_ has stopped on cellulose fiber and is present in the samples. In this region, there are two peaks at 1163 cm^−1^ and 990 cm^−1^, which can be attributed to the cellulose signals due the C-O stretching vibration at 1163 cm^−1^ and C=C bending at 990 cm^−1^ [40]. Additionally, there are two small peaks located at 1128 and 1045 cm^−1^ for all four composite samples and for pure TiO_2_. These signals can be attributed to the vibration mode of Ti-OH and Ti-O-C band, respectively [41,42,43]. Given all above, the main difference between reference and composites samples is the occurrence of an intense signal in 400–800 cm^−1^, which is characteristic for TiO_2_.

**Figure 8:**
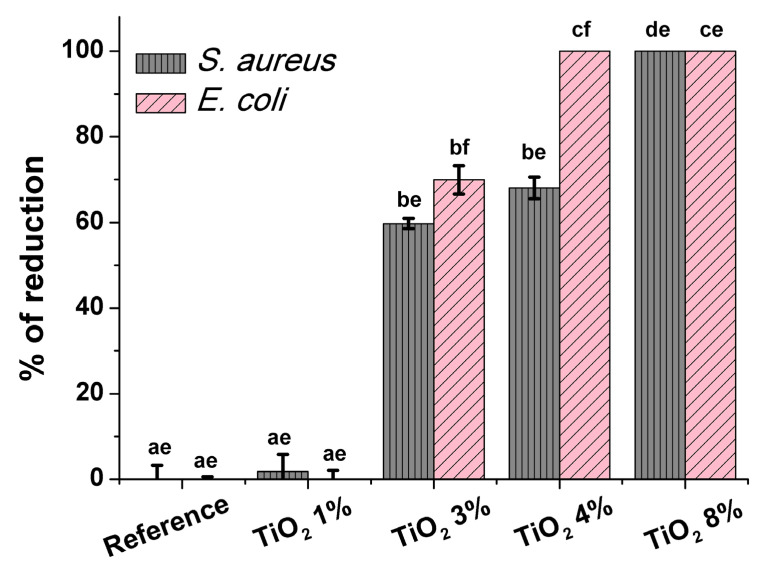
The antibacterial properties of functionalized paper after 24 h of incubation. Obtained data are presented as the mean of the percent of reduction compared to control ± standard error of the mean (SEM). Values with different letters are significantly different (*P* < 0.05): a, b, c, d—statistically significant differences between the samples; e, f—statistically significant differences between the strains.

The antibacterial properties of prepared samples were determined by absorption method according to the ISO 20743. For this analysis, the reference and four composite samples were tested against two representative microorganisms: *Escherichia coli* and *Staphylococcus aureus*. Both strains are common pathogens causing toxic shoch syndrome, skin infection, and endocarditis infection for humans and animals. They can also be found in water, soil, and environmental pollutions [44,45]. The results of antibacterial activity of prepared composts can be found in Figure 8. A detailed description is presented in the experimental section. The viable bacterial counts recovered from the fabrics after 24 h of contact time are shown in Table 2. The reference samples did not cause any bacterial killing during the 24 h contact time; therefore, it is not shown in the Figure 8.

As can be seen from Figure 8, each of the samples exhibit significant antibacterial properties. Percent of reduction for Sample Paper 3 wt% TiO_2_, Sample Paper 4 wt% TiO_2_, and Paper 8 wt% TiO_2_ for *E. coli* after 24 h of incubation is equal to 70%, 100%, and 100%, respectively. A surprisingly strong difference of percent of reduction value between samples containing 3 wt% and 4 wt% TiO_2_ can be observed, despite a slight change of TiO_2_ content in the samples. For *S. aureus* bacteria, the percent of reduction for Samples 1, 2, and 3 is 60%, 68%, and 100%, respectively. Overall, bacteriostatic effect for cellulose fibers modified with titanium dioxide was observed.

It is clearly seen that cellulose/TiO_2_ composites show much better antibacterial properties against *E. coli* than against *S. aureus*. This is due to the differences in the structure of the cell walls of these bacteria. Gram-positive bacteria (e.g., *Streptococcus pneumoniae*, *Streptococcus aureus*) contain a thick peptidoglycan layer in the cell wall, whereas Gram-negative bacteria (e.g., *Escherichia coli*) possess a thin layer of peptidoglycan. Additionally, for Gram-negative bacteria, an outer membrane consisting of a lipopolysaccharide is present [46]. The construction of the cell wall of Gram-positive bacteria can increase the cell resistance against Ti ions, while the structure of Gram-negative bacteria favors an affinity in the adherence of the Ti ion to the cell membrane [5]. The above features result in better cellulose/TiO_2_ composites’ action against *E. coli*.

## 3. Materials and Methods

### 3.1. Materials

Titanium dioxide (TiO_2_) was supplied from Grupa Azoty Police (Police, Poland). Cellulose pulp (density ~40 g/dm^3^) consisting of short eucalyptus-derived fibers and long wood-derived fibers was received from Arctic Paper S.A. (Kostrzyn and Odrą, Poland).

### 3.2. Preparation of the Cellulose Paper Sheet with TiO_2_

The sheets were prepared with a mixture of two kind of fibers: (i) eucalyptus-derived pulp and (ii) wood-derived cellulose pulp. The grammage of obtained paper sheets was 80 g/m^2^. Firstly, the homogeneous solutions of TiO_2_ were prepared. For this purpose, a suitable amount of TiO_2_ corresponding to 1, 3, 4, and 8 wt% were sonicated in water for 30 min. Next, 45 g of eucalyptus-derived pulp and 55 g of wood-derived cellulose pulp were mixed together by mechanical stirrer. Then, the suspensions were added to cellulose pulp and mechanically mixed for 10 min. Appropriate paper sheets were as-formed by Rapid-Köthen Automatic Sheet Forming Machine in accordance to PN-ISO 5262-2. The control paper, without TiO_2_, was prepared with the same procedure, excluding incorporation of TiO_2_ suspension.

### 3.3. Characterization

Transmission electron microscopy (Tecnai F20-based at 200 kV accelerating voltage, Thermo Fisher Scientific, Waltham, MA, USA) was used to characterize the structure of used filler and to calculate particles’ size distribution based on diameter measurements of 100 nanoparticles. The morphology of prepared paper sheets was observed by scanning electron microscope (SEM) (TESCAN, VEGA 3, acquired in the 30 kV acceleration voltage, TESCAN, Brno, Czech Republic) preceded by deposition of 15 nm-thin layer of chromium to avoid the illumination and destruction of samples. The phase composition was determined by X-ray diffraction (XRD) patterns by using a Empyrean (Malvern Panalytical, Malvern, UK) diffractometer using Cu Kα radiation. Thermogravimetric analysis was carried out using TA-Q600 SDT TA Instrument (TA Instrument, New Castle, DE, USA) under the air atmosphere at ramping rate of 10 °C/min from room temperature to 900 °C. Raman spectra were determined on inVia Raman Microscope (RENISHAW, New Mills Wotton-under-Edge, UK) with an excitation wavelength of 785 nm. Fourier transform infrared spectroscopy (FTIR) was used to determine the chemical bonding in the samples. All the absorption spectra were recorded on Nicolet 6700 FT-IR Spectrometer (Thermo Scientific, USA).

### 3.4. Antimicrobial Properties Characterization

#### 3.4.1. Microorganisms and Cultivation Conditions

The antimicrobial properties of functionalized paper and the control sample were determined using Gram-negative bacteria *Escherichia coli* ATCC 8739 (American Type Culture Collection) and Gram-positive bacteria *Staphylococcus aureus* subsp. *aureus* ATCC 6538.

Prior to the experiment, the *S. aureus* and *E. coli* were plated onto the BHIA (Brain Heart Infusion agar, BioMaxima, Poland) and cultivated overnight at 37 °C. After the incubation, one colony of each microorganism was transferred to 10 mL of BHI and incubated overnight in the same culture conditions while shaking.

#### 3.4.2. Determination of Antibacterial Activity

Antimicrobial activity was evaluated according to ISO 20743:2013 Textiles—determination of antibacterial activity of textile products with slight modifications. Before the measurements, all samples were irradiated with UV light for 15 min. Briefly, the 0.4 g of functionalized paper and control sample were transferred to Petri dish. Next, 200 µL of bacterial suspension in concentration 3 × 105 CFU/mL in BHI medium was applied in several points on each tested sample and incubated for 24 h at 37 °C. After incubation, the samples were transferred into 50 mL plastic tubes (3.8 cm diameter) (Polypropylene Conical Centrifuge Tube, Becton Dickinson and Company, USA) with 20 mL of phosphate-buffered saline (PBS, Sigma Aldrich, Germany) and vortexed for 30 s in order to remove the bacterial cells adsorbed on the surface of the samples. The number of bacterial cells was determined by quantitative plating on BHI agar medium after 24 h of incubation at 37 °C.

## 4. Conclusions

A simple and scalable technique of obtaining cellulose paper functionalized with titanium dioxide nanoparticles is presented. The morphology of prepared composites was investigated by SEM and showed that TiO_2_ had deposited on the surface of cellulose fibers. The actual value of fillers in a paper disk was determined with combustion method in accordance to ISO 1762:2001. The results showed content of TiO_2_ in composites equal to 3.62%, 3.75%, and 8.40%. Composition of prepared samples was confirmed by various methods, such as Raman Spectroscopy, XRD, or FTIR. All paper composites have been tested towards antibacterial properties. Percent of reduction of *E. coli* and *S. aureus* bacteria showed satisfactory results. Results showed up to 100% bacteria removal, which together with properties such as scalable technique and easy availability of components, gives the potential for industrial applications.

## Figures and Tables

**Table 1:** Data obtained from TGA analysis and International Organization of Standard (ISO) ash content for all samples.

Sample	Ash According to ISO 1762:2001	Mass at 525 °C According to TGA
Reference	0.13	2.39
Paper TiO_2_ 1%	1.70	2.57
Paper TiO_2_ 3%	3.62	2.88
Paper TiO_2_ 4%	3.75	3.02
Paper TiO_2_ 8%	8.40	10.50

**Table 2:** Viable bacterial counts (colony-forming unit (CFU)/mL) after 24 h contact time showing log reduction in treated sample compared to untreated sample.

		Bacterial Counts (CFU/mL)		% Reduction	
**After 24 h contact time**	**Sample**	***S. aureus***	***E. coli***	***S. aureus***	***E. coli***
	Reference	1.7 × 10^7^ ± 2.9 × 10^6^	1.1 × 10^7^ ± 1.0 × 10^6^	-	-
	Paper TiO_2_ 1%	1.4 × 10^7^ ± 2.6 × 10^6^	1.1 × 10^7^ ± 3.6 × 10^6^	1.75 ± 1.25	0.08 ± 0.64
	Paper TiO_2_ 3%	8 ± 10^2^ ± 51.54	1.4 × 10^2^ ±76.2	59.75 ± 0.38	69.94 ± 1.04
	Paper TiO_2_ 4%	2 × 10^2^ ± 24.96	0 ± 0	68.07 ± 0.79	100 ± 0
	Paper TiO_2_ 8%	0 ± 0	0 ± 0	100 ± 0	100 ± 0

## Data Availability

Data are available with authors. Further inquiries can be directed to the corresponding author.

## References

[1] Geng H.J., Yuan Z.W., Qin M.H. (2013). Dissolving Approach of Cellulose and its Application in Producing Cellulose Functional Material. Adv. Mater. Res..

[2] Lee K.-Y., Aitomäki Y., Berglund L.A., Oksman K., Bismarck A. (2014). On the use of nanocellulose as reinforcement in polymer matrix composites. Compos. Sci. Technol..

[3] Dash M., Chiellini F., Ottenbrite R. (2011). Chitosan—A versatile semi-synthetic polymer in biomedical applications. Prog. Polym. Sci..

[4] Tavakolian M., Okshevsky M., Van De Ven T.G.M., Tufenkji N. (2018). Developing Antibacterial Nanocrystalline Cellulose Using Natural Antibacterial Agents. ACS Appl. Mater. Interfaces.

[5] Arularasu M.V., Harb M., Sundaram R. (2020). Synthesis and characterization of cellulose/TiO_2_ nanocomposite: Evaluation of in vitro antibacterial and in silico molecular docking studies. Carbohydr. Polym..

[6] Edgar K.J., Zhang H. (2020). Antibacterial modification of Lyocell fiber: A review. Carbohydr. Polym..

[7] Pintarić L.M., Škoc M.S., Bilić V.L., Pokrovac I., Kosalec I., Rezić I. (2020). Synthesis, Modification and Characterization of Antimicrobial Textile Surface Containing ZnO Nanoparticles. Polym..

[8] Oun A.A., Shankar S., Rhim J.-W. (2020). Multifunctional nanocellulose/metal and metal oxide nanoparticle hybrid nanomaterials. Crit. Rev. Food Sci. Nutr..

[9] Bischoff N.S., De Kok T.M., Sijm D.T., Van Breda S.G., Briedé J.J., Castenmiller J.J., Opperhuizen A., Chirino Y.I., Dirven H., Gott D. (2020). Possible Adverse Effects of Food Additive E171 (Titanium Dioxide) Related to Particle Specific Human Toxicity, Including the Immune System. Int. J. Mol. Sci..

[10] Pinget G., Tan J., Janac B., Kaakoush N.O., Angelatos A.S., O’Sullivan J., Koay Y.C., Sierro F., Davis J., Divakarla S.K. (2019). Impact of the Food Additive Titanium Dioxide (E171) on Gut Microbiota-Host Interaction. Front. Nutr..

[11] Zhang X., Xiao G., Wang Y., Zhao Y., Su H., Tan T. (2017). Preparation of chitosan-TiO_2_ composite film with efficient antimicrobial activities under visible light for food packaging applications. Carbohydr. Polym..

[12] Alizadeh-Sani M., Mohammadian E., McClements D.J. (2020). Eco-friendly active packaging consisting of nanostructured biopolymer matrix reinforced with TiO_2_ and essential oil: Application for preservation of refrigerated meat. Food Chem..

[13] Yu X., Lu G., Ye J.S., Peng H., Ye J., Dang Z. (2019). Degradation of tris-(2-chloroisopropyl) phosphate via UV/TiO_2_ photocatalysis: Kinetic, pathway, and security risk assessment of degradation intermediates using proteomic analyses. Chem. Eng. J..

[14] Motora K.G., Wu C.-M., Xu T.-Z., Chala T.F., Lai C.-C. (2020). Photocatalytic, antibacterial, and deodorization activity of recycled triacetate cellulose nanocomposites. Mater. Chem. Phys..

[15] Li Y., Tian J., Yang C., Hsiao B.S. (2018). Nanocomposite Film Containing Fibrous Cellulose Scaffold and Ag/TiO_2_ Nanoparticles and Its Antibacterial Activity. Polymers.

[16] Chauhan I., Mohanty P. (2015). In situ decoration of TiO_2_ nanoparticles on the surface of cellulose fibers and study of their photocatalytic and antibacterial activities. Cellulose.

[17] Yang B., Chen Y., Shi J. (2019). Reactive Oxygen Species (ROS)-Based Nanomedicine. Chem. Rev..

[18] Riente P., Noël T. (2019). Application of metal oxide semiconductors in light-driven organic transformations. Catal. Sci. Technol..

[19] Zhu L., Ali A., Shu Y., Ullah K., Cho K.-Y., Oh W.-C. (2015). Detection of reactive oxygen species (ROS) and investigation of efficient visible-light-responsive photocatalysis via nanoscale PbSe sensitized TiO_2_. Sep. Purif. Technol..

[20] Raza M.A., Kanwal Z., Rauf A., Sabri A.N., Riaz S., Naseem S. (2016). Size- and Shape-Dependent Antibacterial Studies of Silver Nanoparticles Synthesized by Wet Chemical Routes. Nanomaterials.

[21] Jeżowska-Bojczuk M., Stokowa-Sołtys K. (2018). Peptides having antimicrobial activity and their complexes with transition metal ions. Eur. J. Med. Chem..

[22] Singh H., Tiwari K., Tiwari R., Pramanik S.K., Das A. (2019). Small Molecule as Fluorescent Probes for Monitoring Intracellular Enzymatic Transformations. Chem. Rev..

[23] Schenzel K., Fischer S. (2001). NIR FT Raman Spectroscopy—A Rapid Analytical Tool for Detecting the Transformation of Cellulose Polymorphs. Cellulose.

[24] Ofem M.I. (2018). Deformation of microfibrillated chitin film and composites. J. Mater. Sci..

[25] Hsieh Y.-C., Yano H., Nogi M., Eichhorn S.J. (2008). An estimation of the Young’s modulus of bacterial cellulose filaments. Cellulose.

[26] Wypych A., Bobowska I., Tracz M., Opasinska A., Kadlubowski S., Krzywania-Kaliszewska A., Grobelny J., Wojciechowski P. (2014). Dielectric Properties and Characterisation of Titanium Dioxide Obtained by Different Chemistry Methods. J. Nanomater..

[27] Frank O., Zukalova M., Laskova B., Kürti J., Koltai J., Kavan L. (2012). Raman spectra of titanium dioxide (anatase, rutile) with identified oxygen isotopes (16, 17, 18). Phys. Chem. Chem. Phys..

[28] Szymańska-Chargot M., Cybulska J., Zdunek A. (2011). Sensing the Structural Differences in Cellulose from Apple and Bacterial Cell Wall Materials by Raman and FT-IR Spectroscopy. Sensors.

[29] Kumar S., Asokan K., Singh R.K., Chatterjee S., Kanjilal D., Ghosh A.K., Asokan K. (2014). Investigations on structural and optical properties of ZnO and ZnO:Co nanoparticles under dense electronic excitations. RSC Adv..

[30] Ijadpanah-Saravy H., Safari M., Khodadadi-Darban A., Rezaei A. (2014). Synthesis of Titanium Dioxide Nanoparticles for Photocatalytic Degradation of Cyanide in Wastewater. Anal. Lett..

[31] Dodoo-Arhin D., Buabeng F.P., Mwabora J., Amaniampong P.N., Agbe H., Nyankson E., Obada D., Asiedu N.Y. (2018). The effect of titanium dioxide synthesis technique and its photocatalytic degradation of organic dye pollutants. Heliyon.

[32] Spurr R.A., Myers H. (1957). Quantitative Analysis of Anatase-Rutile Mixtures with an X-Ray Diffractometer. Anal. Chem..

[33] Oliveira R.L., Vieira J.G., Barud H.S., Assunção R.M.N., Filho G.R., Ribeiro S.J.L., Messadeqq Y. (2015). Synthesis and Characterization of Methylcellulose Produced from Bacterial Cellulose under Heterogeneous Condition. J. Braz. Chem. Soc..

[34] Chen W., He H., Zhu H., Cheng M., Li Y., Wang S. (2018). Thermo-Responsive Cellulose-Based Material with Switchable Wettability for Controllable Oil/Water Separation. Polymers.

[35] Plermjai K., Boonyarattanakalin K., Mekprasart W., Phoohinkong W., Pavasupree S., Pecharapa W. (2019). Optical Absorption and FTIR Study of Cellulose/TiO_2_ Hybrid Composites. http://epg.science.cmu.ac.th/ejournal/.

[36] Karimi S., Feizy J., Mehrjo F., Farrokhnia M. (2016). Detection and quantification of food colorant adulteration in saffron sample using chemometric analysis of FT-IR spectra. RSC Adv..

[37] Abderrahim B., Abderrahman E., Mohamed A., Fatima T., Abdesselam T., Krim O. (2015). Kinetic Thermal Degradation of Cellulose, Polybutylene Succinate and a Green Composite: Comparative Study. World J. Environ. Eng..

[38] Gomes J., Lincho J., Domingues E., Quinta-Ferreira R.M., Martins R. (2019). N–TiO_2_ Photocatalysts: A Review of Their Characteristics and Capacity for Emerging Contaminants Removal. Water.

[39] Rajakumar G., Rahuman A.A., Roopan S.M., Khanna V.G., Elango G., Kamaraj C., Zahir A.A., Velayutham K. (2012). Fungus-mediated biosynthesis and characterization of TiO_2_ nanoparticles and their activity against pathogenic bacteria. Spectrochim. Acta Part A Mol. Biomol. Spectrosc..

[40] Liang P., Chen C., Zhao S., Ge F., Liu D., Liu B., Fan Q., Han B., Xiong X. (2013). Application of Fourier Transform Infrared Spectroscopy for the Oxidation and Peroxide Value Evaluation in Virgin Walnut Oil. J. Spectrosc..

[41] Bensaha R., Bensouy H. (2012). Synthesis, Characterization and Properties of Zirconium Oxide (ZrO_2_)-Doped Titanium Oxide (TiO_2_) Thin Films Obtained via Sol-Gel Process. Heat Treatment—Conventional and Novel Applications.

[42] Kalaiarasi S., Jose M. (2016). Streptomycin loaded TiO_2_ nanoparticles: Preparation, characterization and antibacterial applications. J. Nanostructure Chem..

[43] Vasconcelos D.C.L., Costa V.C., Nunes E.H.M., Sabioni A.C.S., Gasparon M., Vasconcelos W.L. (2011). Infrared Spectroscopy of Titania Sol-Gel Coatings on 316L Stainless Steel. Mater. Sci. Appl..

[44] Bannerman D.D., Paape M.J., Lee J.-W., Zhao X., Hope J.C., Rainard P. (2004). *Escherichia coli* and *Staphylococcus aureus* Elicit Differential Innate Immune Responses following Intramammary Infection. Clin. Diagn. Lab. Immunol..

[45] Tong S.Y., Davis J.S., Eichenberger E., Holland T.L., Fowler V.G. (2015). *Staphylococcus aureus* Infections: Epidemiology, Pathophysiology, Clinical Manifestations, and Management. Clin. Microbiol. Rev..

[46] Slavin Y.N., Asnis J., Häfeli U.O., Bach H. (2017). Metal nanoparticles: Understanding the mechanisms behind antibacterial activity. J. Nanobiotechnol..

